# Self-Regulation in Informal Workplace Learning: Influence of Organizational Learning Culture and Job Characteristics

**DOI:** 10.3389/fpsyg.2021.643748

**Published:** 2021-03-10

**Authors:** Anne F. D. Kittel, Rebecca A. C. Kunz, Tina Seufert

**Affiliations:** Abt. Lehr-Lernforschung, Universität Ulm, Ulm, Germany

**Keywords:** self-regulation, informal workplace learning, learning strategies, mastery-approach goal orientation, self-efficacy, job characteristics, organizational learning culture

## Abstract

The digital shift leads to increasing changes. Employees can deal with changes through informal learning that enables needs-based development. For successful informal learning, self-regulated learning (SRL) is crucial, i.e., to set goals, plan, apply strategies, monitor, and regulate learning for example by applying resource strategies. However, existing SRL models all refer to formal learning settings. Because informal learning differs from formal learning, this study investigates whether SRL models can be transferred from formal learning environments into informal work settings. More precisely, are all facets relevant, and what are the relational patterns? Because informal workplace learning occurs through interaction with the context, this study investigates the influence of context, i.e., organizational learning culture and job characteristics (autonomy, task identity, and feedback) on SRL. Structural equation modeling of *N* = 170 employees in various industries showed the relevance of the self-reported metacognitive strategies planning, monitoring, and regulation; the resource strategies help-seeking and effort regulation; and deep processing strategy elaboration. However, there was no evidence for organization strategies. The learning strategies were associated with self-efficacy and mastery-approach goal orientation. Regarding context, results supported indirect effects over self-motivational beliefs of learning strategies. Organizational learning culture was connected with mastery-approach goal orientation, whereas job characteristics autonomy and feedback were related to self-efficacy, which were again related to SRL strategies. Therefore, context can empower employees not only to accomplish their tasks but to develop themselves by applying SRL strategies. The results are discussed, and practical implications are outlined.

## Introduction

Digitalization leads to more changes that are rapid in the workplace (Bell and Kozlowski, 2008). To deal successfully with these changes, informal learning in the workplace is crucial (Segers et al., 2018). During informal learning, employees can develop themselves based on their needs (Cunningham and Hillier, 2013; Noe et al., 2013; Manuti et al., 2015; Segers et al., 2018), for example, when learning how to use new software to resolve a specific task. In contrast, formal professional development often does not adequately address the individual need for development because it usually provides knowledge based on isolated skills. For successful informal learning, self-regulated learning (SRL) is central (Vancouver et al., 2017), i.e., to control one’s learning process by setting and planning goals and applying, monitoring, and regulating learning strategies (Panadero, 2017). However, the existing SRL models all relate primarily to formal educational settings like school or university. Therefore, the question arises as to whether these models can be transferred to workplace learning, which comprises some rather formal learning opportunities but mainly also rather informal learning situations? What are the differences between formal and informal learning settings? In formal learning, learning situations are characterized by a high degree of structure, external validation, a classroom setting and trainer control (Segers et al., 2018). Outcomes are knowledge and general skills (Tynjälä, 2008). In informal learning, learning occurs mainly through interaction and participation in a context. There is a low degree of structure, no external validation, a workplace settings and learner control (Segers et al., 2018). More various learning activities are used, often involving a variety of tools, resulting in primarily situation-specific skills and tacit knowledge (Tynjälä, 2013). In educational settings as well as at the workplace both forms of learning can be observed. However, whereas in educational settings the emphasis is more on formal learning opportunities, workplace learning is characterized by an emphasis on informal learning (Cunningham and Hillier, 2013; Noe et al., 2013; Tynjälä, 2013). Although some studies have investigated SRL across different educational contexts (e.g., Palalas and Wark, 2020), they did not investigate in depth, if in mainly informal workplace learning SRL aspects are used likewise as described in formal learning environments. So, one can ask in view of the differences: Can the SRL models be transferred from formal learning environments to the informal work setting despite the differences? Do all facets play a role, and what relational patterns can be found?

Informal workplace learning occurs through interaction and participation in the work context, i.e., as a by-product of completing certain tasks (Tynjälä, 2013). However, it is not clear how the work context is specifically related to SRL. This is the second research question of the study, how the work context relates to SRL. On the one hand, organizational learning culture considers if employees are encouraged and rewarded for learning (Marsick and Watkins, 2003). If the learning culture is perceived as positive, employees should be more motivated to learn in a self-regulated manner. On the other hand, the activity during which employees learn can be investigated more closely by looking at their respective job characteristics (Hackman and Oldham, 1976): Do employees have the opportunity to learn something new? Can activities be processed as a whole? Is the feedback helpful? These characteristics might limit or enable the extent to which employees can learn and whether they are motivated to use SRL strategies.

Informal learning has gained increasing attention, especially over the last two decades. It has been claimed that human resources management should shift from job-based to competency-based training, which encompasses a greater variety of learning opportunities and explicitly includes informal learning activities (Segers et al., 2018). Informal learning is usually defined on continua in comparison to formal learning activities. On the one hand, formal learning activities have a high degree of structure, an external validation, a classroom setting, are trainer-controlled, and involve an external stimulus. On the other hand, informal learning activities have a low degree of structure, no external validation, no classroom setting, and the learning stimulus is internal (Segers et al., 2018). Although these continua endings describe key features of informal or formal learning situations, learning situations often involve formal and informal aspects. They might have complementary features, for example formal learning situations might involve informal learning activities like discussion during breaks (Segers et al., 2018). SRL is crucial for learning success for formal learning (e.g., Sitzmann and Ely, 2011; Panadero, 2017), as well for informal learning: Because informal learning is mainly learner controlled (e.g., Noe et al., 2013), successful self-regulation is crucial for success (Vancouver et al., 2017).

### Self-Regulated Learning

Self-regulated learning is defined as “self-generated thoughts, feelings, and actions that are planned and cyclically adapted to the attainment of personal goals” (Zimmerman, 2000, p. 14). Various models describe SRL in formal education; however, there is a different focus on either the processes or the structure of SRL (Panadero, 2017). Yet, all models emphasize that learners first examine the task, set goals, and plan their learning based on their motivational beliefs. Then, learners apply various learning strategies and monitor whether they are reaching their goals, and regulate their learning (Puustinen and Pulkkinen, 2001; Panadero, 2017). Therefore, learning strategies are crucial in all models, and their application is closely connected with motivational beliefs. Furthermore, context, which is crucial for workplace learning, is also part of many SRL models (Panadero, 2017) and, therefore, will also be examined in greater depth.

#### Self-Regulated Learning Strategies

Goal setting often triggers further SRL strategies (Locke and Latham, 2002; Sitzmann and Ely, 2011; Panadero, 2017), which are differentiated into cognitive, metacognitive, and resource learning strategies (Pintrich et al., 2000). *Cognitive strategies* are essential for successfully processing concrete learning content and, therefore, for acquiring, storing, and retrieving relevant information. Examples include the relatively shallow surface strategy, *rehearsal* (i.e., learning through repetition), the more advanced deep processing strategy, *organization* (i.e., structuring key information into meaningful units), or *elaboration* (i.e., connecting information from multiple sources or with prior knowledge; Pintrich, 2004). *Metacognitive strategies* are crucial to the metacognitive control of SRL and to the effective use of cognitive strategies. They encompass *planning* (i.e., making a learning plan and choosing cognitive strategies based on the goals, requiring forethought), *monitoring* (i.e., monitoring whether the applied strategies are sufficient to reach the goals), and *regulation* (i.e., changing the learning behavior if problems arise while monitoring, such as applying different strategies; Zimmerman, 2002). *Resource strategies* support learning further by managing the learning environment and external resources (e.g., help-seeking or time management; Pintrich, 1999).

#### Motivational Beliefs

To begin the SRL process and set learning goals, motivational beliefs, i.e., goal orientation or self-efficacy, are crucial. They are included in most SRL theories, and they are most prominent in Zimmerman’s model (Zimmerman, 2002; see also Sitzmann and Ely, 2011; Panadero, 2017 for an overview). Research has shown the special relevance of mastery (-approach) goals (Elliot et al., 2017) in contrast to performance goals. Learners with a mastery goal orientation engage in more SRL strategies and can regulate their learning according to their goals (e.g., Pintrich, 1999; Payne et al., 2007; De Clercq et al., 2013). Therefore, on the one hand, this study focuses on mastery-approach goal orientation. On the other hand, according to theoretical models and studies on SRL (Sitzmann and Ely, 2011), the expectation of self-efficacy, that is to say, having confidence in one’s ability to complete tasks, is an essential success factor for SRL. More precisely, self-efficacy means “judgments of how well one can execute courses of actions required to deal with prospective situations” (Bandura, 1982, p. 122).

#### Context

Finally, SRL is also influenced by external factors like the context. Context is a relevant factor in most current SRL models (e.g., Zimmerman, 1989; Boekaerts and Corno, 2005; Hadwin et al., 2011; Winne, 2011; for more details see the review of Panadero, 2017). According to these models, the context can influence SRL in different ways. For example, the Socially Shared Regulated Learning model (SSRL; Hadwin et al., 2011) especially stresses the role of collaborative learning and, thereby, the social environment as a context factor. In contrast, the COPES (conditions, operations, products, evaluations and standards) model (Winne, 2011) refers to the conditions required to adapt to task demands during SRL. These conditions can be specified by the resources, the instructional cues, the time, and the social context. Consequently, there are different ways in which the context influences SRL. However, few research studies have investigated context effects in SRL despite SSRL empirically (Panadero, 2017), in formal education as well as especially in informal learning settings.

The question arises as to whether these models of SRL and their most basic assumptions can be transferred to informal workplace learning. As there are several differences between formal learning and informal workplace learning, there might also be differences in self-regulation in these settings.

### Differences Between Informal Workplace Learning and Formal Learning

Informal workplace learning differs in several aspects from formal education. The two methods can be distinguished according to the 3-P Model of Workplace Learning, devised by Tynjälä (2013) as *presage*, considering the learner and the learning context, the *process* in terms of learning activities, and the learning outcomes (i.e., the *product*). The following distinction describes key features of formal and informal learning situations. However, many learning activities are also partly formal as well as partly informal or may complement each other, for example, informal learning might lead to participation in formal training, or formal training is accompanied by individual or collaborative informal learning activities (Segers et al., 2018).

#### Learner and Context

Individual presage factors, such as prior knowledge and experience, are important, influential factors for learning in informal workplace learning, as they are for formal education. However, in the workplace, additional important factors such as agency, commitment, or life situation may have a different impact whether employees engage in learning activities (Tynjälä, 2013).

In the informal workplace, learning is not the primary concern of employees. Instead, workplace learning is a side effect of work. It results from participation in the context of work, the second presage factor. Employees’ interpretation of context factors frames the direction in which individuals can develop themselves and how they can do so. Context factors encompass, among other things, organization of work, organizational climate, and manager support (Tynjälä, 2013). In formal educational settings, the teaching context also matters, how the classroom is organized or which instructional design the teacher applies (Biggs, 1999; Tynjälä, 2013). The individual has limited options to alter learning objectives, usually based on a curriculum (Tynjälä, 2008).

#### Learning Activities

In formal education, the learning process, and hence the learning activities, are often based on individual cognitive mental activities, which are usually abstract and aimed at the acquisition of explicit knowledge, generalized skills, and principles (Resnick, 1987; Tynjälä, 2008). In contrast, in informal workplace learning, learning-related activities are more diverse, ranging from doing the job itself to reflecting one’s work experiences, collaboration, or tackling in new challenges (Tynjälä, 2013). They often require collaboration with other people because work outcomes are often team-based. Formal education also often includes collaborative learning activities (e.g., see for more details the SSRL model, Hadwin et al., 2011), although tests are taken often individually, requiring primarily individual learning activities. Informal workplace learning use a wider variety of tools because tool use is not prohibited as often in formal education. Because informal learning occurs in the workplace, it includes contextualized reasoning, which leads to implicit and tacit knowledge, and situation-specific competencies (Resnick, 1987; Tynjälä, 2008).

The structure of learning content, support by others, time and objectives is high in formal learning, whereas in informal learning, learning is less structured, requiring learners to engage autonomously in learning (Segers et al., 2018).

In formal learning, the learning stimulus is often external, that is set by a curriculum or an instructor (Segers et al., 2018), and is externally validated by taking tests to require certificates, for which learners use specific strategies like repetition. In contrast, in informal learning, the learning stimulus is internal, for example dissatisfaction with current ways of acting (Segers et al., 2018). Therefore, the primary emphasis in formal education is on knowledge acquisition, whereas the emphasis in workplace learning is on work (Tynjälä, 2013).

#### Learning Outcomes

These various learning activities in informal workplace learning lead to more diverse learning products or outcomes. They extend from academic performance to task performance to personal development, i.e., learning as personal goal, team performance, identity, role performance, organizational benefits (such as improved productivity), and they include ineffective working practices (Tynjälä, 2013). By contrast, in formal education, these outcomes are often prescribed due to test questions more limited to quantitative (e.g., facts or skills), qualitative (e.g., structure, transfer), and affective (e.g., involvement) learning outcomes (Biggs, 1999; Tynjälä, 2013). Yet, recent research investigates also different conceptions of learning success in formal education, such as success of previously defined goals (Guterman, 2020).

All things considered, learning occurs quite differently during mainly informal workplace learning regarding learners, context, learning activities, and learning outcomes. Given these differences, the following question arises: Can the SRL models be transferred from formal learning environments to the informal work context despite their differences? Do all facets play a role, and what are the relational patterns?

### Implications for Self-Regulated Learning in Informal Workplace Learning

First of all, the use of SRL aspects can also vary across different formal learning contexts (e.g., Broadbent, 2017). Although there are differences in how learning occurs between formal and informal learning settings, there is no initial reason to assume that the SRL process as a whole proceeds differently. The process implies that learning is planned according to motivational beliefs, learning strategies are applied, monitored, and regulated, e.g., by applying resource strategies, and that the context influences SRL. The importance of context is one main difference from formal learning settings because workplace learning occurs through interaction and participation in the work context (Tynjälä, 2013). However, it is not clear how the specific learning context, i.e., the work context in informal workplace learning, is specifically related to SRL. In the following, the implications for SRL in informal workplace learning are further outlined regarding SRL strategies, motivational beliefs, and context.

#### Self-Regulated Learning Strategies

Given that the SRL process is comparable to formal settings in workplace learning, the metacognitive strategies should also be relevant in informal learning, as well as the application of resource strategies. However, learning objects, learning context, and the people involved must be adapted to the informal context. Also, regarding cognitive strategies, it is questionable whether the same learning strategies can be used in informal learning. This is because certain procedures and processes are specific to the learning setting. For example, formalized exams or specified worksheets are typical in the formal setting and do not occur in an informal setting. Therefore, strategies aimed only at these situations, such as surface strategies, e.g., repeating for exams, are not directly transferable to the informal setting. Moreover, as these surface strategies turned out to be ineffective (e.g., Richardson et al., 2012) – we focused our analysis on deep learning strategies.

#### Motivational Beliefs

It is to be expected that motivational orientation also plays an essential role in the informal context, as described in SRL models. We focus here on stable motivational orientations and not on the current state of motivation because our focus is on the general learning processes in informal workplace learning. Motivational beliefs (i.e., goal orientation or self-efficacy) are crucial when beginning the SRL process and setting the learning goals. We assume that these motivational beliefs are similarly associated with SRL strategies in formal settings.

The preceding discussion of the implications of SRL in informal workplace learning is based on theoretical assumptions. However, there are also some empirical studies that have investigated SRL in workplace learning. Although research mainly investigated SRL in formal workplace settings (see Sitzmann and Ely, 2011 for a meta-analysis of formal workplace training), a few, mostly qualitative studies, have examined SRL in informal workplace learning. In general, they report that fewer sub-processes are relevant for SRL in informal workplace learning (van Eekelen et al., 2005; Schulz and Stamov Roßnagel, 2010; Margaryan et al., 2013; Milligan et al., 2015). One study found evidence for the relevance of only three SRL aspects of informal workplace learning, i.e., task interest/value, task strategies, and self-evaluation (Milligan et al., 2015). However, another study showed the importance of metacognitive SRL strategies and mastery-approach goal orientation, whereas cognitive strategies were not relevant (Schulz and Stamov Roßnagel, 2010).

#### Learning Context in Informal Workplace Learning

In general, according to several SRL models, the learning context influences different aspects of SRL, such as motivation or learning strategies (e.g., Panadero, 2017).

Previous studies investigating SRL in different contexts have defined context diversely: from a rather narrow definition as the subject in higher education (Rotgans and Schmidt, 2009) or by testing background knowledge of participants, for example in MOOCs if participants have a background associated with the learning topic or not (Hood et al., 2015). Some studies define context somehow broader by comparing online vs. blended learning (e.g., Broadbent, 2017) or face to face vs. online (Lee and Tsai, 2011), whereas some have a more general view by investigating formal vs. informal (mobile) learning settings (Palalas and Wark, 2020). However, they did only investigate if mobile learning enhances SRL and vice versa, but did not differentiate in depth SRL in informal or formal learning settings. Moreover, some studies define context more overarching. For example, a study investigated in formal education classroom context factors like classroom time (Raver et al., 2012). One study investigated in the clinical workplace social, contextual, and individual context factors (van Houten-Schat et al., 2018), finding that time pressure, patient care and supervision enabled and hindered SRL. Although the results may be typical for the clinical context, one can derive that in the workplace the context varies in many ways from formal educational settings (e.g., Tynjälä, 2013). Several implications for SRL can be derived, with possible differences in comparison to typical educational settings and potential context factors in informal workplace learning and how they might relate with SRL.

In general, learning context is highly relevant in informal workplace learning because learning is understood as the result of an interaction with the context (Tynjälä, 2013). According to the 3-P model, the relevant context factors in the workplace are organizational structure, organization of work, HR development, staff expertise, organizational climate, manager support, collaborative climate, orientation toward learning and innovations, partnerships, and networks (Tynjälä, 2013). These factors may vary in different organizations or even in organizations or teams because, crucially, individual perceptions of these factors will differ. Consequently, the employee’s interpretation of the context is crucial for their engagement in learning activities (Tynjälä, 2013).

From the perspective of workplace training research, potentially influencing contextual factors in the workplace are also diverse. In their recent review of about 100 years of training and development research, Bell et al. (2017) found that context is a main factor of training success. They proposed a system perspective, where actions before and after learning influence how employees learn and feel (Bell et al., 2017). More precisely, they differentiated three factors that can enhance or hinder development: (a) supervisor and peer support, (b) positive climate (which is related to motivation to learn; see the meta-analysis of Colquitt et al., 2000), and (c) work experiences (Bell et al., 2017). Peer support, as one facet of the first factor, is usually part of SRL models (e.g., SSRL model, Hadwin et al., 2011) when, for example, collaborative learning or help-seeking as a resource strategy are included (e.g., Pintrich, 1999). Therefore, this factor is applicable to educational settings as well as to the workplace. As other factors are more exclusively relevant in the workplace, we wanted to particularly address their relevance to SRL. We, therefore, combine positive climate and supervisor support as factors that constitute the organizational learning culture. Work experiences are based on the assumption that employees individually perceive their respective work characteristics. They can be specified as Job Characteristics (Hackman and Oldham, 1976) in which the most relevant facets of motivating and positively experienced workplaces are described. Some empirical studies specify the relations SRL has with the organizational learning culture and job characteristics.

##### Organizational learning culture and self-regulated learning

An organizational learning culture promotes organizational learning and adaptation; it structures the environment to support learning at all organizational levels (Marsick and Watkins, 2003; Watkins and Kim, 2018). On individual level, a learning culture ideally creates continuous learning opportunities, encourages dialogue, promotes cooperation, and enables coworkers to have a collective vision. On structural level, a learning culture can provide both leadership and systems for recording and exchanging learning content. The structural level makes it possible to convert individual learning into collective organizational knowledge and to increase the financial performance of the organization (Watkins and Marsick, 1993). Consequently, a learning culture defines specific values and results to encourage the employees to gain new knowledge and skills (Marsick and Watkins, 2003; Pantouvakis and Bouranta, 2017). Hence, establishing a learning culture should create a learning enhancing environment and encourage employees to engage in (self-regulated) learning activities.

Empirical research associates learning culture with engagement in informal learning activities (Nurmala, 2014). Regarding SRL strategies, some studies have shown that organizational learning culture is connected with SRL strategies. However, this approach has yet to be conceptualized in line with current SRL models (e.g., “workplace learning,” Clarke, 2005 or “deep learning approach,” Froehlich et al., 2014). Yet, if SRL were to be conceptualized according to current models, some recent studies have suggested positive relations between the learning enhancing environments and SRL strategies (Schulz and Stamov Roßnagel, 2010; Milligan et al., 2015; Littlejohn et al., 2016). However, regarding the influence of provided learning opportunities, the results were mixed. For example, Milligan et al. (2015) found a positive relation to SRL strategies (cf. Schulz and Stamov Roßnagel, 2010). One study found evidence for a moderating effect of training climate, a concept quite similar to learning culture, on goal orientation: A good training climate combined with a high mastery-approach goal orientation led to more self-reported learning success of recent informal learning episodes. Therefore, an established learning culture and a high mastery-approach goal orientation interact and positively influence learning success (Schulz and Stamov Roßnagel, 2010). These results suggest a rather distal, indirect impact of organization learning culture on informal learning activities. Yet, results were slightly heterogeneous, because only managerial and job support together, but neither of them alone, were moderating the relationship of mastery-approach goal orientation on learning success. This might be due to the homogenous sample investigated. However, there were no similar effects for SRL strategies (Schulz and Stamov Roßnagel, 2010).

Regarding motivation, relatively few studies have investigated its relation to mastery-approach goal orientation or self-efficacy. There are hints that learning culture seems to be slightly positively associated with mastery-approach goal orientation (Joo and Park, 2010) and self-efficacy (Simosi, 2012).

Consequently, there are some indications that organizational learning culture might be related to SRL strategies, goal orientation, and self-efficacy. More research is needed to examine these relationships more thoroughly.

##### Job characteristics and self-regulated learning

According to the highly influential (e.g., Parker et al., 2017). Job Characteristics model, five job characteristics (skill variety, task identity, task significance, autonomy, and feedback) affect several work outcomes positively (motivation, which again affects job performance, satisfaction, performance, absenteeism, and turnover; Hackman and Oldham, 1976; see Noe et al., 2014; Parker et al., 2017). The job characteristic *autonomy* refers to the degree of freedom in carrying out a job (Hackman and Oldham, 1975), concerning work scheduling, decision making, and work methods (Morgeson and Humphrey, 2006; Stegmann et al., 2010). *Task identity* is defined by the degree to which a job comprises a complete service or product. This complete piece of work has a visible outcome, and, therefore, results can be easily identified (Hackman and Oldham, 1975). *Feedback* means whether the employees receive feedback on their task (Hackman and Oldham, 1975); feedback can be from the task or from others (Morgeson and Humphrey, 2006).

In contrast, skill variety (perception of necessary skills and competencies for tasks) and task significance (importance of the task for others) rely mainly on individual perceptions. Moreover, in the 3-P model of workplace learning, the relevant learning context factor, organization of work, can be specified by autonomy, task identity, and feedback, not by skill variety or task significance (Tynjälä, 2013). Therefore, we only focused on autonomy, task identity, and feedback as relevant factors for SRL.

Autonomy can restrict or empower employees regarding what they learn and how (i.e., choosing their learning task, planning, conducting the necessary steps, and the possibilities for regulation). Task identity was found to enhance the learning capacities provided by a job (Dehnbostel, 2018). Completing a piece of work in its entirety should foster an understanding of the task and help one choose the strategy accordingly. Feedback is a source of self-efficacy (Bandura, 2010) that could constitute an event showing employees the need to change their approach and to develop themselves.

There is a lack of empirical research on SRL strategies (Parker et al., 2017). Although some studies found that job characteristics can enhance SRL (see the review by Wielenga-Meijer et al., 2010; Gijbels et al., 2014; Noe et al., 2014; Lin et al., 2018), they do not investigate the relation to SRL in detail because they do not measure SRL according to current SRL model frameworks. Regarding motivation, a task accompanied by high autonomy and feedback leads to higher motivation (Parker et al., 2017). However, few studies have investigated the relation to mastery goal orientation and self-efficacy. Job characteristics seem to positively affect self-efficacy (Wang and Netemeyer, 2002; van Mierlo et al., 2006). In short, there is a clear need for more research regarding job characteristics and their relationship with SRL.

### Summary of Hypotheses

Overall, the paper aims to analyze whether basic assumptions of SRL models, which mainly refer to formal learning settings, can be transferred to informal workplace learning and how the special learning context in informal workplace learning relates to SRL. Therefore, the two main aims are (1) Can SRL models be transferred to the informal work setting, i.e., do all facets play a role, and what are the relational patterns? (2) How does the context influence SRL in informal workplace learning?

Regarding the first aim, we use current models and empirical results on SRL, mainly referring to formal educational settings, as a starting point. We investigate which learning strategies can be adapted to informal workplace learning. We assume the following learning strategies to be relevant for informal workplace learning: metacognitive strategies (Hypothesis 1a), resource strategies (Hypothesis 1b), and cognitive deep processing strategies (Hypothesis 1c). Furthermore, we analyze which relational patterns can also be found in informal workplace learning. For motivational beliefs, we assume a relation between mastery-approach goal orientation and learning strategies (Hypothesis 2a) and between self-efficacy and learning strategies (Hypothesis 2b).

Regarding the second aim, we expect a connection between learning context and SRL as assumed by SRL models. On the one hand, the organizational learning culture is positively associated with SRL (Hypothesis 3a), and, on the other hand, the job characteristics autonomy, task identity, and feedback are positively associated with SRL (Hypothesis 3b).

## Materials and Methods

### Participants

We conducted a questionnaire-based online survey to address the research questions. We addressed employees of different companies in Germany and Austria. Participants could also take part via paper and pencil questionnaires. Requirements for participation were as follows: being currently employed or self-employed and being fluent in the German language. Participation was voluntary and anonymous. All participants signed an informed consent form prior to the survey. In accordance with German legislation, institutional review board approval is not required for this type of study. This study complies with human subjects guidelines of national research committees as well as the APA Ethics Code Standards.

The heterogeneous sample consisted of 170 participants (50% women) from various industries, sizes of organizations, and educational levels. Participants were aged from 18 to 66 years (*M* = 37.25, *SD* = 12.98). Company branches differed as well: most participants worked in service companies (25%), followed by industry (16%), energy (14%), technology (11%), health (9%), and educational institutions (9%). Also, the size of the organizations varied: one-third of the participants worked in small companies with a maximum of 50 employees (30.2%), one-third worked in medium to large companies with 50 to 1,000 employees (34.3%), and one-third worked in organizations with more than 1,000 employees (35.5%). They worked in various working sectors, such as in business administration (15%) and business related services like human resources (19%) or sales and marketing (14%), in research and development (14%) or IT-sector (14%), in personal services (8%) or production of goods (5%). Some participants (30.6%) reported more than 15 years of work experience, whereas another third had less than 3 years of work experience. Most participants were employed, and some were in leading positions (22%). The participants had a rather high level of education: 58% had a university degree, 40% had vocational education, and only 2.3% had no vocational education. Overall, the sample was representative of the German population because it matched the general distribution, according to official German statistics (Bundesagentur für Arbeit, Statistik/Arbeitsmarktberichterstattung, 2019).

### Measures

To address the hypotheses, we used well-established self-report scales, which are described in the following. Table 1 shows confirmatory factor analyses (CFA) results and the Cronbach’s alpha for each scale. To minimize construct-unrelated variance of errors (Harvey et al., 1985), participants were asked to rate the items on a 5-point Likert scale (1 = *not true at all*, 5 = *totally true*) throughout the survey.

**TABLE 1:** Result of confirmatory factor analysis and Cronbach’s alpha of the study variables without SRL strategies.

Variable	CFA result	Cronbach’s alpha
Mastery-approach goal orientation	Saturated model*	α = 0.82
Self-efficacy (10 items)	χ^2^(35) = 84.691, CFI = 0.881, RMSEA (90% CI) = 0.092 [0.068; 0.115], SRMR = 0.065	α = 0.80
Self-efficacy (6 items)	χ^2^(9) = 13.271, CFI = 0.981, RMSEA (90% CI) = 0.053 [0.000; 0.105], SRMR = 0.033	α = 0.80
Organizational learning culture	χ^2^(14) = 11.307, CFI = 1.00, RMSEA (90% CI) = 0.000 [0.000; 0.055], SRMR = 0.030	α = 0.82
Job characteristics as second-order factor with subfactors autonomy (methods, decisions); autonomy (planning); feedback from others; task identity	χ^2^(98) = 143.373, CFI = 0.958, RMSEA (90% CI) = 0.052 [0.034; 0.068], SRMR = 0.049	Job characteristics (α = 0.88): autonomy in methods and decisions (α = 0.88); autonomy in planning (α = 0.87): feedback from others (α = 0.74); task identity (α = 0.75)

*Because the CFA for mastery-approach goal orientation revealed a saturated model fit, we conducted an additional CFA for both motivational beliefs mastery-approach goal orientation correlated with self-efficacy (6-item scale), which are connected according to a meta-analysis (Huang, 2016). Results revealed an excellent fit: χ^2^(36) = 32.277, CFI = 0.983, RMSEA (90% CI) = 0.038 [0.000; 0.072], SRMR = 0.047.

#### Self-Regulated Learning Strategies in the Workplace Setting

In order to assess the above mentioned SRL strategies in relatively informal learning scenarios in the workplace, we developed a self-report scale based on current SRL models (Panadero, 2017), Pintrich’s learning strategy taxonomy (1999), and an established scale (MSLQ; Pintrich et al., 1991). We adapted existing items for better alignment to the work setting. Instead of learning in class, we asked for learning at work; instead of study material or tasks, we used work material or tasks. We also changed the term *students* to *colleagues*, and *instructor* to *manager*, if applicable. We did not adapt more general aspects, like learning for tests or marking a text. Additionally, we created new items focusing specifically on the informal work setting and on specific work situations, such as meetings. The scale comprised subscales based on common taxonomies: for cognitive deep processing strategies organization and elaboration (Pintrich, 1999); for metacognitive strategies planning, monitoring, and regulation; and for resource-oriented strategies, help-seeking, and effort regulation.

Two experts reviewed the scales before the questionnaire was piloted. Then, the items were revised for improved clarity (see Supplementary Tables 1, 2 for precise wording and details). We used CFAs and model comparison to examine the internal structure of the item pool, and to investigate the model fit. We excluded all items with loadings ≤0.4 (Hulland, 1999) to improve model fit. The results of CFA and model comparison are presented in the results section because they address the first hypothesis. As a result of these analyses, we created second-order factor SRL strategies (α = 0.77), comprising elaboration with four items (α = 0.58), planning with five items (α = 0.67), monitoring and regulation with five items (α = 0.71), effort regulation with two items (α = 0.71), and help-seeking with three items (α = 0.51).

#### Mastery-Approach Goal Orientation

We assessed mastery-approach goal orientation with three items of the Patterns of Adaptive Learning Scales (PALS; Midgley et al., 2000). The items were adapted as described above and translated into German (for precise wording, see Supplementary Table 3).

#### Self-Efficacy

We used six items from the German version of the short version of the Generalized Self-Efficacy Scale (Schwarzer and Jerusalem, 1999) to assess self-efficacy. As the CFA with ten original items revealed a poor fit with partly low loadings (≤0.4), we excluded the four items with loadings ≤0.4 (see Supplementary Table 4). The scale with six items revealed an excellent fit (see Table 1).

#### Learning Culture

We assessed the learning culture of the organization with the short version of the Dimensions of the Learning Organization Questionnaire (DLOQ, Watkins and Marsick, 1997; Yang, 2003), which consists of seven items. CFA revealed an excellent fit (see Table 1). As there was no German version of the DLOQ, we carefully translated the items (for the exact wording, see Supplementary Table 5).

#### Job Characteristics

We used the German Version of the Work-Design-Questionnaire (WDQ, Morgeson and Humphrey, 2006) from Stegmann et al. (2010) to measure job characteristics. We aimed to assess autonomy in methods, in decisions, and in planning; feedback from others and feedback from the task, and task identity. CFA revealed identification problems for the separate factors, autonomy in methods and autonomy in decisions, and for the two feedback factors. Therefore, we used six items to assess the combined autonomy factors for methods and decisions. Furthermore, we assessed feedback from others, henceforth referred to as feedback, with three items. We also assessed autonomy in planning and task identity using three and four items. A second-order factor model revealed an excellent fit (Table 1).

### Rationale for Analyses

First, we conducted CFAs for all scales to evaluate the measurement models of the constructs. We dropped items with loadings ≤0.4 (Hulland, 1999). We used CFA and structural equational modeling (SEM) to address our research questions, investigating the latent relationships between the constructs. This statistical method allows for complex relations between latent variables and measurement errors (Byrne, 2013).

We used CFA and SEM to address our first research question regarding the structure of SRL in informal workplace learning. For the first hypothesis concerning the learning strategies for SRL in the workplace, we compared different models by including different SRL strategies (i.e., different second-order factor models and correlated models). For the second hypothesis concerning the relation to motivational beliefs, we used SEM. We included motivational beliefs (i.e., mastery-approach goal orientation and self-efficacy) into the models that resulted in the first hypothesis. We also used SEM to analyze the second research question about how the context influences SRL in the workplace. We expanded the resulting model of the first research question by including the contextual factors of organizational learning culture and job characteristics with the subfactors autonomy of methods and decisions, autonomy in planning, feedback, and task identity. In a second step, we investigated all four job characteristics, respectively, by investigating four models, each including a distinct job characteristic but being otherwise the same.

We used various fit indices to evaluate the model fit of the factor and structural equation models (West et al., 2012): Chi-Square test, the Tucker–Lewis fit index (TLI), the comparative fit index (CFI), the root mean square error of approximation (RMSEA) with its 90% confidence interval, and the standardized root mean square residual (SRMR). Missing data were accounted for by using the full information maximum likelihood estimator (FIML). We used the robust maximum likelihood estimator (MLR) to deal with normality issues.

All analyses were conducted using R version 1.1.447. We used *lavaan* (Version 0.6–5; Rosseel, 2012) for CFA and SEM.

## Results

### Preliminary Analyses

We examined the means, standard deviations, and correlations of the study variables to gain the first insights into our research questions (see Table 2). In general, employees reported relatively high use of SRL strategies, especially for elaboration, whereas their use for monitoring and regulation was less frequent. They also reported slightly above average mastery-approach goal orientation and self-efficacy. The employees perceived their context very differently, as is apparent in the higher standard deviations. In particular, organizational learning culture and feedback from others had a lower mean in comparison to perceived autonomy. Correlational analysis showed positive relations between SRL strategies and motivation and between learning culture and job characteristics.

**TABLE 2:** Means, standard deviations, and correlations between study variables.

Variables	*MW* (*SD*)	1.	2.	3.	4.	5.	6.	7.	8.	9.	10.	11.	12.	13.
1. Mastery-approach GO	3.77 (0.74)	–	0.214**	0.062	0.226**	0.375***	0.269***	0.226**	0.374***	0.308***	0.166*	0.072	0.243**	0.148
2. Self-efficacy	3.56 (0.52)		–	0.156*	0.208**	0.301***	0.150	0.318***	0.366***	0.215**	0.319***	0.184*	0.277***	0.208**
3. Elaboration	4.21 (0.45)			–	0.257**	0.204**	0.131	0.256**	0.549***	0.225**	0.282***	0.228**	0.070	−0.010
4. Planning	3.84 (0.59)				–	0.353***	0.251**	0.105	0.634***	0.276***	0.306***	0.239**	0.207**	0.193*
5. Monitoring and regulation	3.72 (0.51)					–	0.356***	0.283***	0.690***	0.189*	0.039	−0.027	0.175*	0.092
6. Help-seeking	3.99 (0.55)						–	0.229**	0.631***	0.176*	0.128	0.065	0.273***	0.187*
7. Effort regulation	4.09 (0.55)							–	0.628***	0.128	0.108	0.118	0.061	0.114
8. SRL strategies	3.97 (0.34)								–	0.314***	0.271***	0.198**	0.251**	0.193*
9. OLC	3.18 (0.70)									–	0.519***	0.385***	0.592***	0.333***
10. Autonomy (M&D)	3.77 (0.76)										–	0.718***	0.368***	0.376***
11. Autonomy (planning)	3.68 (0.93)											–	0.272***	0.237**
12. Feedback from others	3.10 (0.82)												–	0.264***
13. Task identity	3.34 (0.77)													–

GO, goal orientation; OLC, organizational learning culture. Autonomy (M&D), autonomy in methods and decisions. N = 170. *p < 0.05, **p < 0.01, ***p < 0.001.

### Self-Regulated Learning in Informal Workplace Learning

To address the first research question as to whether SRL can be conceptualized in informal workplace learning similarly to formal learning settings, we first investigated the SRL strategies in informal workplace learning by comparing different CFAs to analyze the first hypothesis.

#### Strategies in Informal Workplace Learning

We first investigated the model fit of a second-order model with all measured SRL strategies as a second-order factor. However, the model demonstrated an unsatisfactory fit (Table 3). To identify which of the separate strategies enhanced the consistency of the model, we used model comparison. We tested six different models with second-order factor SRL strategies, which comprised all but one SRL strategy, to systematically compare them with the first model that had an unsatisfactory fit. The model comparison significantly supported a second-order model with SRL strategies as a second-order factor with subfactors elaboration and planning, a shared factor for monitoring and regulation,^1^ effort regulation, and help-seeking without organization as a subfactor (see Table 3 model 2b). Also, a correlated model with the same strategies showed a similar fit. Consequently, the results suggest that not all learning strategies in informal workplace learning contribute to SRL. Therefore, the results only partially support the first set of hypotheses: hypothesis 1a regarding metacognitive strategies, and hypothesis 1b, regarding resource strategies, are supported, whereas hypothesis 1c, regarding deep processing strategies, is only partially supported.

**TABLE 3:** Results of confirmatory factor analysis and model comparisons of SRL strategies.

Model	CFA result	Model comparison to model 1
*Model 1.* SRL strategies as second-order factor with subfactors elaboration; organization; planning; monitoring and regulation; help-seeking; effort regulation	χ^2^(224) = 295.544, CFI = 0.888, RMSEA (90% CI) = 0.043 [0.029; 0.056], SRMR = 0.071	AIC: 8715; BIC: 8950.2
*Model 2a.* SRL strategies as second-order factor with all subfactors as Model 1 apart from elaboration	χ^2^(147) = 204.023, CFI = 0.894, RMSEA (90% CI) = 0.048 [0.031; 0.063], SRMR = 0.070	AIC: 7442; BIC: 7635.4; *Δχ*^2^(77) = 91.605, *p* = 0.122
*Model 2b.* SRL strategies as second-order factor with all subfactors as Model 1 apart from organization	χ^2^(147) = 137.075, CFI = 1.000, RMSEA (90% CI) = 0.000 [0.000; 0.028], SRMR = 0.057	AIC: 6979; BIC: 7170.7; *Δχ*^2^(77) = 0.754, *p* < 0.001
*Model 2c.* SRL strategies as second-order factor with all subfactors as Model 1 apart from planning	χ^2^(130) = 168.200, CFI = 0.913, RMSEA (90% CI) = 0.042 [0.021; 0.058], SRMR = 0.068	AIC: 6655.9; BIC: 6841.0; *Δχ*^2^(94) = 91.605, *p* = 0.012
*Model 2d.* SRL strategies as second-order factor with all subfactors as Model 1 apart from monitoring and regulation	χ^2^(130) = 180.979, CFI = 0.885, RMSEA (90% CI) = 0.048 [0.030; 0.064], SRMR = 0.068	AIC: 6984.9; BIC: 7160.9; *Δχ*^2^(94) = 114.72, *p* = 0.072
*Model 2e.* SRL strategies as second-order factor with all subfactors as Model 1 apart from help-seeking	χ^2^(165) = 242.480, CFI = 0.882, RMSEA (90% CI) = 0.049 [0.034; 0.063], SRMR = 0.073	AIC: 7559; BIC: 7802.8; *Δχ*^2^(59) = 62.367, *p* = 0.357
*Model 2f.* SRL strategies as second-order factor with all subfactors as Model 1 apart from effort Regulation	χ^2^(184) = 257.100, CFI = 0.877, RMSEA (90% CI) = 0.048 [0.034; 0.061], SRMR = 0.071	AIC: 7979.6; BIC: 8192.8; *Δχ*^2^(40) = 37.147, *p* = 0.599
*Model 3.* Correlated SRL strategies elaboration, planning, monitoring and regulation, help-seeking, effort regulation	χ^2^(142) = 131.187, CFI = 1.000, RMSEA (90% CI) = 0.000 [0.000; 0.027], SRMR = 0.054	AIC: 6979.2; BIC: 7189.3; *Δχ*^2^(82) = 166.46, *p* < 0.001

*Model comparison supported Model 2b and Model 3.*

#### Relation Between Self-Regulated Learning Strategies and Motivational Beliefs in Informal Workplace Learning

**FIGURE 1:**
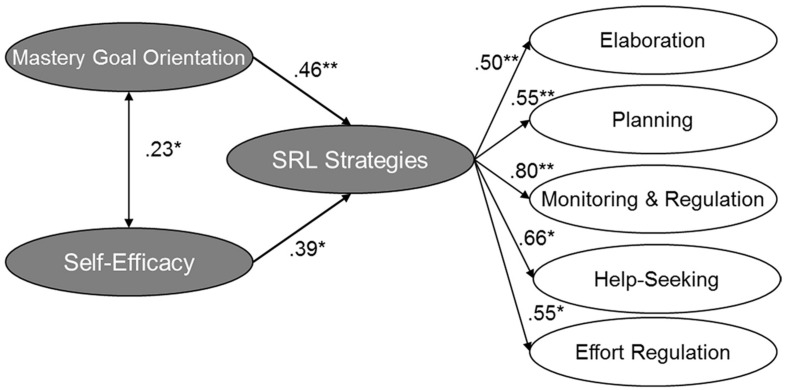
Structural equation model showing the relations of mastery-approach goal orientation and self-efficacy to SRL strategies. Coefficient is significant at the 0.05 level *, at the 0.01 level **. Paths which are n.s. are gray colored. *N* = 170. The model fit is χ^2^(342) = 400.801, CFI = 0.939, RMSEA (90% CI) = 0.032 [0.016; 0.044], SRMR = 0.064.

**FIGURE 2:**
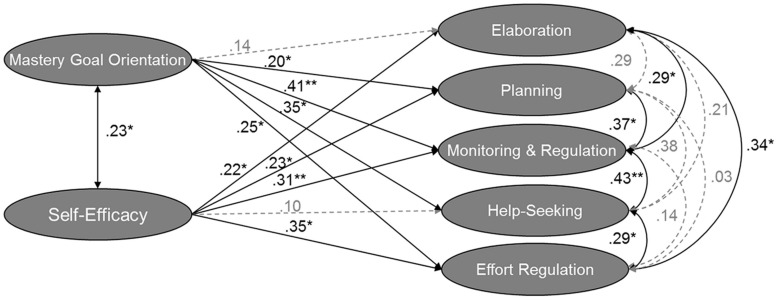
Structural equation model showing the relations of self-efficacy and mastery-approach goal orientation to correlated SRL strategies. Coefficient is significant at the 0.05 level *, at the 0.01 level **. Paths which are n.s. are gray colored. *N* = 170. The model fit is χ^2^(329) = 387.514, CFI = 0.940, RMSEA (90% CI) = 0.032 [0.016; 0.044], SRMR = 0.061.

As a next step, we investigated the relation of SRL strategies with motivational beliefs to analyze the second set of hypotheses. For this, we added the relations of mastery-approach goal orientation and self-efficacy to SRL strategies into the previously supported model 2b with second-order factor SRL strategies. Model fit demonstrated a good fit (see Figure 1). As expected, both motivational beliefs were moderately to strongly related to the SRL strategies, supporting the second set of hypotheses. For a more in-depth investigation of the relations between learning strategies and motivational beliefs, we analyzed a second alternative model (see Figure 2) without a second-order factor for SRL strategies, using the second supported model from hypothesis 1, i.e., model 3, which also revealed a very good model fit. We found that the metacognitive strategies and effort regulation were both related to mastery-approach goal orientation and self-efficacy. Elaboration was only significantly related to self-efficacy, whereas help-seeking was only related to mastery-approach goal orientation. These results supplement the previous results. The second hypotheses are only partially supported because not all the strategies were related to the two motivational factors.

Consequently, concerning the first research question, we found a close connection of mastery-approach goal orientation and self-efficacy with most SRL strategies in informal workplace learning, consisting of elaboration, planning, monitoring and regulation, and help-seeking strategies.

### The Influence of Context on Self-Regulated Learning in Informal Workplace Learning

**FIGURE 3:**
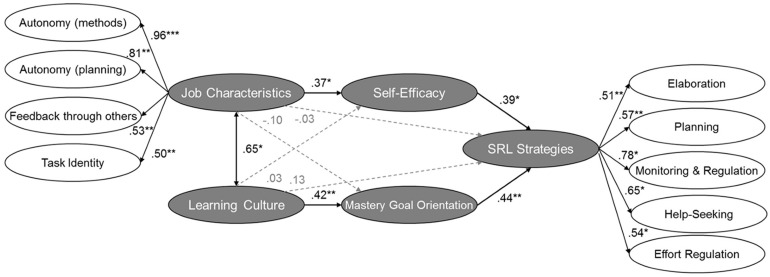
Structural equation model showing the relations of the job context (organizational learning culture and job characteristics) to mastery-approach goal orientation, self-efficacy, and SRL strategies. Coefficient is significant at the 0.05 level *, at the 0.01 level **, at the 0.001 level ***. Paths which are n.s. are gray colored. *N* = 170. The model fit is χ^2^(206) = 1605.411, CFI = 0.861, RMSEA (95%CI) = 0.044 [0.038; 0.050], SRMR = 0.080.

We used SEM to analyze the second research question as to whether the context, i.e., the organizational learning culture and the job characteristics autonomy in methods and decision, autonomy in planning, feedback, and task identity, are connected with SRL. We modeled the context variable learning culture correlated with job characteristics as a second-order factor. We incorporated the context variables into the previous model applied in hypothesis 2 with second-order factor SRL strategies and motivational beliefs. The model included the contextual variables learning culture and job characteristics, mastery-approach goal orientation, self-efficacy, and SRL strategies, and demonstrated a good fit (see Figure 3). Contrary to our expectations, we found only indirect effects of context over self-motivational beliefs on SRL strategies in the workplace (see Figure 3). Learning culture was strongly associated with mastery-approach goal orientation, which was, in turn, related to SRL strategies. Also, for the job characteristics autonomy in methods and decision, autonomy in planning, feedback, and task identity, we only found an indirect relation to SRL strategies. The characteristics were directly related to self-efficacy, which was then related to SRL strategies. Therefore, we found only partial evidence for the third hypotheses.

We conducted mediation analyses for this model to investigate these indirect relationships in greater depth. For organizational learning culture, the indirect relationship was significant (indirect effect over mastery goal orientation = 0.188, *p* = 0.015, total effect: = 0.320, *p* = 0.069). However, it failed to reach significance for job characteristics (indirect effect over self-efficacy = 0.145, *p* = 0.096, total effect: = 0.116, *p* = 0.557), with a surprisingly negatively direct effect, which was, however, not significant.

Although a second-order factor of job characteristics is theoretically reasonable according to the Job Characteristics model, the complete model, including all job characteristics, is not supported, and single job characteristics have been researched separately or in different constellations (Parker et al., 2017). There could be an assumption that different job characteristics might differently impact self-efficacy and SRL strategies. Therefore, we estimated four models in a second step, which included only one job characteristic in each model, but, otherwise, comprised the same variables as in the full model (Figure 3). Therefore, we can differentiate the results for the different job characteristics. We only found indications of mediation for autonomy in methods, decision, and feedback over self-efficacy, which, however, failed to reach significance (see Table 4). In contrast, autonomy in planning and task identity was not significantly related to self-efficacy, nor to SRL strategies, and there was no indication of mediation.

**TABLE 4:** Results of mediation analysis for different job characteristics.

Mediation model including specific job characteristics	Model parameters: effects of job characteristic			Mediation analysis: effects of job characteristic		Model fit
				
	→ SE	→ SRL strategies	SE → SRL strategies	Indirect effect over SE	Total effect on SRL strategies	
Autonomy (methods)	0.334*	−0.043	0.397*	0.132*	0.090	χ^2^(689) = 888.381, CFI = 0.895, RMSEA (90% CI) = 0.041 [0.033; 0.049], SRMR = 0.074
Autonomy (planning)	0.135	0.011	0.385*	0.052	0.063	χ^2^(651) = 809.202, CFI = 0.905, RMSEA (90% CI) = 0.038 [0.029; 0.046], SRMR = 0.072
Feedback through others	0.41^*a*^	0.008	0.380*	0.155^*b*^	0.163	χ^2^(651) = 805.874, CFI = 0.900, RMSEA (90% CI) = 0.037 [0.028; 0.045], SRMR = 0.070
Task identity	0.183	0.030	0.383**	0.070	0.100	χ^2^(688) = 806.852, CFI = 0.920, RMSEA (90% CI) = 0.032 [0.022; 0.042], SRMR = 0.070

N = 170. Mediations tested for models, which include, instead of a second-order factor “job characteristics” only a single job characteristic, but are otherwise similar to the full model (see Figure 3). Standardized values are reported. Coefficient is significant on the 0.05 level *, at the 0.01 level **, or the 0.001 level ***. ^a^p = 0.054; ^b^p = 0.118.

## Discussion

This study investigates whether theoretical assumptions of SRL can be transferred from formal learning settings to informal workplace learning because the two settings differ regarding learners, contexts, learning activities, and outcomes. The results of this study indicate that, with some exceptions, several major assumptions of formal learning settings can be adapted to the workplace. Furthermore, the study demonstrates the importance of context in informal workplace learning. Context has an indirect influence on SRL strategies via mastery-approach goal orientation and self-efficacy.

### Self-Regulated Learning in Informal Workplace Learning

The differences in informal learning in the workplace, in comparison to formal settings, seem to impact SRL in informal workplace learning. Results suggest that although the general SRL process seems to be similar, there are implications for informal learning contexts, especially regarding cognitive learning strategies. Some procedures, specific to formal learning settings like formalized exams or prepared worksheets, cannot be transferred to informal workplace learning. Accordingly, strategies aimed at formal settings cannot be directly transferred.

#### Self-Regulated Learning Strategies

We found indications that fewer SRL strategies seem to be relevant for informal workplace learning compared to common SRL models or strategy taxonomies. These results match previous studies, showing that fewer SRL-subprocesses are relevant in informal workplace learning (van Eekelen et al., 2005; Schulz and Stamov Roßnagel, 2010; Margaryan et al., 2013; Milligan et al., 2015). However, it might be possible that further SRL strategies, which we have not addressed in our study, might be important for successful informal workplace learning, such as critical thinking or concentration (see for a comprehensive list Richardson et al., 2012). Future studies might address a broader range of strategies and their impact. Further, it is important that also in different formal learning setting, not every strategy is relevant (e.g., Cleary et al., 2012).

For cognitive strategies, we found the expected evidence for deep processing strategies. This is not surprising because employees apply elaboration strategies (e.g., they try to find resemblances to previous problems) instead of just memorizing specific topics to resolve a task. However, surprisingly, we found no support for organization strategies. Also, previous studies did not support organization strategies as a part of SRL in the workplace (e.g., Schulz and Stamov Roßnagel, 2010). This is surprising because employees often need to organize different tasks in their work environment, as demonstrated in current competency models (e.g., see the industry 4.0 competency model of Prifti et al., 2017). For example, employees structure their tasks and manage their processes. Therefore, organization is part of their work tasks. However, unexpectedly, organization strategies do not seem to be part of their workplace learning. On the one hand, this could be due to employees’ individual understanding of learning. Perhaps they only view organization strategies as part of their task, but not as their workplace learning. Consequently, the employees’ actual understanding of workplace learning is an interesting question, which could be analyzed in further studies. More general, it might be a general issue in workplace learning that employees might not be aware that they are even learning. Maybe a learning culture, which acknowledges explicitly the huge role of employee’s everyday informal workplace learning might initiate a change. On the other hand, perhaps the items we used and adapted failed to assess crucial aspects of organization strategies in the workplace. We used items aimed at the organization of learning material (e.g., notetaking at meetings, ensuring that all materials are available, or writing short instructions). On a more general note, the items were derived based on measurements with a cognitive focus. However, the learning activities in informal workplace learning are more diverse than the mainly cognitive formal learning activities. A measurement, taking these different learning activities into account, might be worthy of consideration. Further, maybe the items were phrased in an unfamiliar way, so employees did not report using that strategy, because they would use a different terminology. This is a familiar measurement problem. For example in research tests claim to measure different constructs, but actually measure the same construct, which represents a jangle fallacy (Kelley, 1927; e.g., academic math self-concept and math self-efficacy; Marsh et al., 2019). Therefore, future research is needed for a more in-depth exploration of the relevance of organization strategies to workplace learning.

As expected, we found evidence for the relevance of metacognitive strategies to SRL in workplace learning. Again, this is in line with previous results (e.g., Schulz and Stamov Roßnagel, 2010 or Milligan et al., 2015, who supported self-evaluation). As in the case of educational settings, the successful application of metacognitive strategies seems crucial. In successful informal workplace learning, employees can deliberately plan, monitor, and regulate their learning activities. Especially monitoring might be crucial for informal workplace learning, because there is often no external source of feedback (Sitzmann and Ely, 2011; Segers et al., 2018). Instead, monitoring is often the only source for employees to recognize, whether they have learned successfully and additionally, if they have achieved their learning goal. In addition, metacognitive reflection during work tasks and processes is crucial for detecting possibilities for their optimization, implying the successful application of SRL. It is possible that, if employees monitor obstacles or barriers in their tasks, they will try to resolve them as a matter of course, thereby trying to regulate them. Employees could autonomously apply different regulation strategies and might have more possibilities than in more prescribed tasks, as in a curriculum in educational settings.

Finally, we found that help-seeking is also relevant to SRL in informal workplace learning. This emphasizes the crucial aspect of collaborative learning, as suggested in the 3-P model (Tynjälä, 2013), and the joint resolving of work problems, which often requires SRL. Effort regulation was also supported as an important SRL factor in workplace learning. Therefore, this result emphasizes the crucial role of the ability to persist when dealing with challenging situations or tasks. As effort regulation was especially associated with elaboration and help-seeking, the study results suggest that employees might try to regulate their efforts by applying other SRL strategies. They could use deep processing cognitive strategies, such as elaboration, or seek help from their colleagues, or look up the relevant information needed to solve a challenging task.

#### Motivational Beliefs Are Related to Self-Regulated Learning Strategies

As expected, we found a medium to strong relationship between mastery-approach goal orientation and self-efficacy and the use of elaboration, metacognitive strategies, help-seeking, and effort regulation strategies. This matches the theoretical assumptions of diverse SRL models about the crucial role of motivation (Panadero, 2017) and has been widely shown in empirical educational research (e.g., the meta-analysis of Richardson et al., 2012). Also, the results of the present study match previous studies, which supported the importance of mastery-approach goal orientation in informal workplace learning (Schulz and Stamov Roßnagel, 2010). In particular, monitoring and regulation, as well as help-seeking, go along with mastery-approach goal orientation, indicating that the entire learning process in relation to work is mostly under the control of the learner. Therefore, learners decide if (and how) they engage in learning activities. This is one of the chief differences between workplace learning and learning in educational settings (Tynjälä, 2013).

More conclusions can be drawn about SRL in informal workplace learning concerning effect sizes in educational settings. For example, in educational settings, there was evidence for a high relation of mastery-approach goal orientation with elaboration (Lin, 2019). In contrast, it was not significant in the present study, which indicates a different impact of mastery-approach goal orientation on SRL strategies in informal workplace learning. In the workplace, it seems that elaboration is not directly linked to mastery-approach goal orientation. Instead, there were indications of a link to self-efficacy, implying that elaboration strategies are more likely to be used after their previous successful use. Also, elaboration strategies were solely related to monitoring and regulation, which indicated that they might be used when the learner reflects on specific tasks. In higher education, elaboration is often fostered through learning tasks and is thus often used by learners who aim to understand the learning itself.

However, out of many other possible motivational factors, we focused on mastery-approach learning goal orientation and self-efficacy because they both play a crucial role in SRL (e.g., Sitzmann and Ely, 2011; Elliot et al., 2017; Panadero, 2017). Future studies could investigate whether our results can be transferred to the 3 × 2 goal orientation framework (Elliot and Hulleman, 2017). Researchers could investigate the influence of other motivational concepts, such as intrinsic motivation, interest, or learners’ self-concept.

### Context Indirectly Enables Self-Regulated Learning in Informal Workplace Learning

One of the most crucial differences between formal and informal workplace learning is constituted by contextual factors. Therefore, we were highly interested in the role of the context in SRL. Contrary to our expectations, we did not find direct effects of context on SRL strategies. However, we found evidence for the indirect effects of contextual factors via motivation. Learning culture was associated with SRL strategies via mastery-approach goal orientation. An organizational learning culture supports the learning and development of employees. It promotes the goal orientation of the employees, encouraging them to strive to understand their learning topic, a process associated with SRL strategies. Job characteristics were associated with SRL strategies via self-efficacy, i.e., job characteristics were related to higher self-efficacy, thereby affecting SRL strategies. More precisely, the results suggest that, in particular, autonomy in methods, decision, and feedback from others related to self-efficacy, whereas autonomy in planning and task identity had no significant indirect effects on SRL strategies via self-efficacy. These results confirm previous studies (e.g., Joo and Park, 2010; Hans and Gupta, 2018), but they contradict other results (e.g., Simosi, 2012; Froehlich et al., 2014; Lin et al., 2018).

However, the study had a cross-sectional design, permitting no causal interpretation. The direction of effects was theoretically grounded by SRL theory, according to which motivational beliefs trigger SRL strategies (e.g., Zimmerman, 2002) and workplace learning assumptions, based on which learners interpret their context and then engage in learning activities (Tynjälä, 2013). Yet, theoretically, different effect directions might be possible. Future, longitudinal studies could investigate if change in contextual factors like the organization’s learning culture or job characteristics causes actually more motivation to learn and more SRL.

This study only analyzed the contextual impact of organizational learning culture and job characteristics. However, the research literature provides several further context factors that could affect SRL in the workplace, such as job demands or job resources (e.g., see the review of Parker et al., 2017), and could be derived, for example, from the job-demands-resources model (Demerouti et al., 2001) or the job demands-control model (van der Doef and Maes, 1999). Such additional context factors should be analyzed in future studies.

**FIGURE 4:**
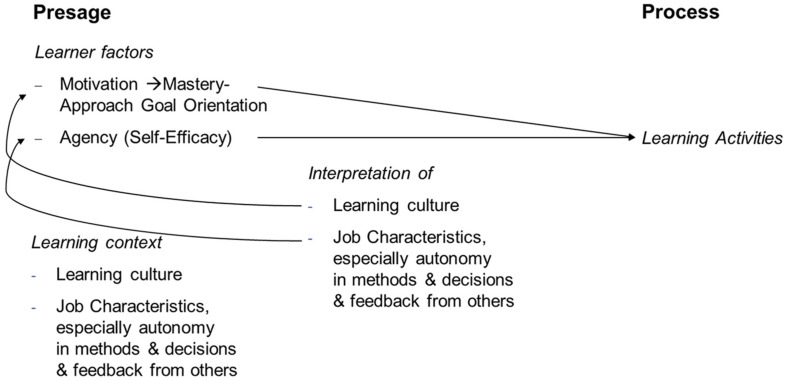
Selected variables of the 3-P model (Tynjälä, 2013) with highlighted study findings. Contrary to expectations, there was no direct path from perception of context to learning activities, only an indirect path over motivation and agency.

In short, these results elaborate on the theoretical assumptions of the 3-P model (Tynjälä, 2013). The presage factor learning context includes organizational climate, manager support, orientation toward learning, and innovations, all of which can be summarized under learning culture. According to the 3-P model, these context factors are interpreted and influenced by the learner. The results of this study show that the perceived learning context factor, learning culture, leads to a higher mastery-approach goal orientation, part of the learner factor motivation in 3-P model terms. Therefore, the model can be complemented by a connection between learning culture and motivation. Also, the presage factor includes the organization of work, which is mainly determined by job characteristics. The empirical results of the study show that, in the 3-P model in particular, perceived autonomy of methods, decision, and feedback from others lead to higher self-efficacy, and therefore, to higher motivation or agency. Therefore, another connection could be incorporated into the model (see Figure 4).

These results represent a detailed exploration of SRL in the workplace. As described above, the results suggest supplementing existing SRL models in the workplace.

### Practical Implications

It is crucial to optimize the potential of every employee to meet the growing demand for professional development to comply with digitalization and other current developments. However, learning in the workplace happens through different activities and with different outcomes than those experienced in formal learning settings. For example, in the workplace, learning is at least 70% informal (Cunningham and Hillier, 2013; Noe et al., 2013; Tynjälä, 2013). One possibility to develop employees beyond their formal workplace training is to strengthen the employees’ abilities to optimize their own development during their work routine (i.e., with informal learning). This could be achieved by helping employees to self-regulate their learning.

On the basis of the results of this study, there are different ways to achieve this aim. First, formal SRL training could be provided. This training could integrate SRL strategies training (i.e., deep processing, metacognitive, and resource strategies) and goal-setting interventions. Furthermore, such training could redefine the understanding of workplace learning. This is crucial because many employees might suppose that learning only takes place in formal workplace learning situations. Such training could modify employees’ understanding of what workplace learning includes. Studies in formal education have shown that SRL training can be effective (e.g., Jansen et al., 2019), but due to the differences between formal and informal learning, there is still a need for studies on informal workplace learning. It is crucial to reduce the gap between training and practice and to provide support for transfer of learning (Blume et al., 2010). Second, SRL assistance could be integrated into the work routine. For example, reflection indications could be integrated on a metacognitive basis, or learning paths could explicitly integrate informal learning phases.

Third, the results of this study show that context plays a crucial role in workplace learning. Therefore, opportunities to indirectly support SRL in the workplace establish an organizational learning culture that supports the employees’ learning, providing feedback (for further details, see the meta-analysis of Kluger and DeNisi, 1996 or see Whitaker and Levy, 2012), and enabling autonomous work. As a consequence, this indirect approach to fostering SRL could be combined with one or more of the direct approaches described above (i.e., formal SRL training or assistance with SRL during the work routine), leading to better SRL in the workplace. This could also shift the focus of professional development toward more than 70% of the informal workplace learning, thereby establishing a more comprehensive understanding of workplace learning.

### Limitations and Future Studies

This study was an attempt to better understand SRL in the workplace by basing assumptions on a more general model of learning in the workplace as compared to educational settings. It focused on the individual and did not take further organizational aspects, such as a multilevel perspective on teams or leadership, into account. These elements would be interesting to investigate in future studies.

Furthermore, this study did not apply process measures for SRL or learning analytics in an actual learning situation. Instead, the aim of the study was to gain a general understanding of SRL in the workplace as a first step. Our applied self-report strategy measurement had a cognitive focus because it was derived from formal educational settings measurements. Self-report relies on introspection, which adults are quite capable of. As a second step, it would be interesting to investigate the concrete learning situations in the workplace in which SRL strategies are applied in practice, and to analyze whether the importance of context remains strong. This would be especially interesting because learners often report different strategies than they actually use and often tend to overestimate their strategy use (Dunlosky and Lipko, 2007). This is because self-report measures are often retrospective and somewhat biased, for example, when monitoring accuracy (Molenaar et al., 2020). However, because learning in the workplace occurs often implicit and unconsciously, actually capturing different learning situations would be challenging. Initially, qualitative research might help to identify these situations. Based on this study’s results, one could start identifying concrete learning situations in work contexts that enable learning, i.e., an encouraging learning culture or job characteristics, and investigate how learners try to achieve their learning goals. Then, in concrete learning situations, one could apply process measures to capture applied SRL strategies and their interplay with motivation and identify how the strategies contribute to a learning outcome. Yet, if learners are learning implicit without awareness that they are learning, process measures might bias results because learners might become aware of their learning activities. However, learning activities in the workplace are more diverse (Tynjälä, 2013), so it would be interesting to measure more precisely which learning strategies are applied in which learning situations.

In addition, this study did not comprise all aspects of SRL suggested by current different SRL models (see Panadero, 2017). Therefore, further studies could also integrate additional motivational aspects (e.g., intrinsic motivation or self-concept) or emotions and investigate them in larger SRL models with a greater sample. They could investigate social learning (SSRL model, Hadwin et al., 2011) in greater depth because we only investigated help-seeking and effort regulation as resource strategies. As learning in the workplace often occurs collaboratively (Tynjälä, 2013), it would be interesting to further explore this aspect of SRL. Future studies could also investigate time management. We suggest that time management strategies might strongly depend on the context because informal workplace learning depends on current tasks and priorities, and especially on the possibility of autonomously managing an employee’s task, and thereby their time. Further research is necessary because the existing results are ambiguous (e.g., see the review of Aeon and Aguinis, 2017).

Finally, the sample was fairly representative according to official German statistics (Bundesagentur für Arbeit, Statistik/Arbeitsmarktberichterstattung, 2019) because it included employees with an equal sex ratio and a wide age range from different educational and hierarchical levels of different industries. Therefore, our results reflect the spectrum of perceived differences in the workplace setting, especially regarding organizational learning culture and job characteristics. In a single company, the variety of these elements would be limited. However, a larger sample would be needed to have more power for the applied SEM statistics and to test for invariance in order to control if results might alter in different company types like manufacturing or sales.

In short, there is a need for more learning that is informal in the workplace to deal with the changes caused by the digital shift. Informal workplace learning has several specificities: it occurs through the interaction of various learning activities with a context resulting in situation-specific skills, whereas formal learning aims at general skill acquisition in highly structured classroom learning settings. Therefore, SRL models, which generally refer to formal educational settings, can only partially be applied to workplace learning. One specificity of informal workplace learning, the significant impact of context, is demonstrated by organizational learning culture and job characteristics autonomy and feedback. These elements motivate and empower employees to use their time at work not only to accomplish their tasks but to further develop themselves by applying the strategies of SRL. This will help employees to keep pace with the increasing changes caused by the digital shift.

## Data Availability Statement

The raw data supporting the conclusions of this article are available from the corresponding author on request.

## Ethics Statement

Ethical review and approval was not required for the study on human participants in accordance with the local legislation and institutional requirements. The patients/participants provided their written informed consent to participate in this study.

## Author Contributions

AK performed conceptualization, methodology, data analysis, writing original draft and editing, and project administration. RK performed conceptualization, methodology, and data analysis. TS performed supervision, conceptualization, and writing review and editing. All authors contributed to the article and approved the submitted version.

## Conflict of Interest

The authors declare that the research was conducted in the absence of any commercial or financial relationships that could be construed as a potential conflict of interest.

## Footnotes

1 We favored a combined factor for monitoring and regulation because it is theoretically reasonable (Pressley and Afflerbach, 1995; Pintrich et al., 2000) and more parsimonious, although the model comparison closely missed significance Δχ^2^(15) = 23.205, *p* = 0.079.
