# Supplementary material for: Self-Regulation in Informal Workplace Learning: Influence of Organizational Learning Culture and Job Characteristics
Source: Front Psychol. 2021 Mar 10;12:643748. doi: 10.3389/fpsyg.2021.643748 (PMC7988190; doi:10.3389/fpsyg.2021.643748)
Supplement: Supplementary file 1 [file Data_Sheet_1.docx]

Supplementary Material

Table S1.

*SRL-questionnaire: used and self-translated English items.*

| Self-translated English items | Used items | Items SRL-model |
| --- | --- | --- |
| **Organization** |  |  |
| When a colleague explains the application of a new system, or something similar to me, I take notes that I can revise before I actually use the system. | Wenn mir ein Kollege die Anwendung eines neuen Systems oder Ähnliches erklärt, mache ich mir Notizen, die ich vor der tatsächlichen Anwendung nochmals durchgehen kann. |  |
| Before I start a new task, I get an overview of my workplace and make sure that I have all information and material needed. | Bevor ich eine neue Aufgabe beginne, verschaffe ich mir einen Überblick über meinen Arbeitsplatz und stelle sicher, dass ich alle nötigen Informationen und Materialien dafür habe. |  |
| In general meetings I take notes of the most important points, so that I can use them for my work later. | In allgemeinen Besprechungen notiere ich mir die wichtigsten Punkte, damit ich sie später für meine Tätigkeit nutzen kann. |  |
| I create short instructions (e.g. for pathways, steps of procedure, click instructions, etc.) for my tasks, to know how to operate in future situations. | Ich erstelle kurze Anleitungen (z. B. für Pfadwege, Arbeitsschritte, Klickanleitungen, etc.) für meine Tätigkeiten, um beim nächsten Mal zu wissen, wie ich vorgehen muss. |  |
| I do not create charts, tables or graphic illustrations in order to facilitate handling my task. | Ich erstelle keine Diagramme, Tabellen oder grafische Darstellungen, um mir die Bearbeitung meiner Aufgabe/ Tätigkeit zu erleichtern. <REVERSED> |  |
| **Elaboration** |  |  |
| While I learn the application of new technologies (e.g. a new software, a new machine, etc.), I try to connect this knowledge with my previous knowledge. | Während ich mir die Anwendung neuer Technik (z. B. eines neuen PC-Programms, einer neuen Maschine, etc.) aneigne, versuche ich, dieses Wissen mit meinem bisherigen Wissen zu verknüpfen. | X |
| When I get new professional information, I try to connect this new knowledge with my previous knowledge. | Wenn ich neue Fachinformationen bekomme, versuche ich das neue Wissen mit meinem bisherigen zu verbinden. | X |
| When I work on a new task, I try to pull together as much information as possible to help me. | Wenn ich eine neue Aufgabe bearbeite, versuche ich alles an Informationen zusammenzutragen was mir weiterhilft. |  |
| When I’m facing a new task or problem, I draw back on previous experience to solve it. | Wenn ich vor einer neuen Aufgabe oder einem Problem stehe, greife ich auf meine bisherigen Erfahrungen zurück, um sie zu lösen. | X |
| I do not create connections between different information I get during a day (e.g. from my supervisor, colleagues, etc.). | Ich stelle keine Zusammenhänge zwischen den verschiedenen (Fach-) Informationen, die ich im Arbeitsalltag bekomme (z. B. über Vorgesetzte, Kollegen, etc.), her. <REVERSED> | X |
| **Planning** |  |  |
| I usually keep track of the tasks that need to be done and establish their sequence. | Ich behalte in der Regel den Überblick über die zu erledigenden Aufgaben und lege eine Reihenfolge dafür fest. | X |
| I do not plan my course of action at work. | Ich plane mein Vorgehen beim Arbeiten nicht. <REVERSED> | X |
| When I start my work, I do not think about how to execute specific tasks. | Ich mache mir zu Arbeitsbeginn keine Gedanken, welche Aufgaben ich wie erledige. <REVERSED> | X |
| I set daily or weekly goals. | Ich setze mir Tages- oder Wochenziele. | X |
| I define for myself, when I want to have specific tasks to be finished. | Ich lege für mich fest, bis wann ich welche Aufgaben erledigt haben möchte. | X |
| **Monitoring** |  |  |
| I check which operational steps I can carry out well and where I could improve myself. | Ich achte darauf, welche Arbeitsschritte ich gut ausführen kann und bei welchen ich mich noch verbessern könnte. | X |
| I regularly make myself aware of the areas where I have improved my skills in and the areas I’m still in need of development. | Ich mache mir regelmäßig bewusst, in welchen Bereichen ich mich verbessert habe und in welchen ich weiterhin Entwicklungsbedarf habe | X |
| I notice when my thoughts get carried away and I’m not focused. | Mir fällt auf, wenn ich mit meinen Gedanken abschweife und nicht ganz bei der Sache bin. |  |
| Before I finish a task, I do not examine if I carried it out correctly / if it is error-free. | Bevor ich eine Aufgabe abschließe, prüfe ich nicht mehr, ob ich diese korrekt ausgeführt habe / ob diese fehlerfrei ist. <REVERSED> |  |
| While carrying out a task, I regularly check if my actions are still constructive. | Beim Bearbeiten einer Aufgabe achte ich regelmäßig darauf, ob meine Vorgehensweise noch zielführend ist. | X |
| **Regulation** |  |  |
| When I’m assigned to a new task, I adapt my function to finish it at its best. | Wenn ich neue Aufgaben bekomme, passe ich meine Arbeitsweise an, um diese bestmöglich fertigzustellen. | X |
| When I find myself losing attention, I try to focus on the task again. | Wenn ich mich dabei ertappe, dass meine Aufmerksamkeit nachlässt, versuche ich mich wieder auf die Aufgabe zu fokussieren. | X |
| When I get a (problem) report e.g. on the computer, and I do not understand it, I read it again slowly and focused to understand it. | Wenn ich eine (Fehler-)meldung z. B. am Computer bekomme und sie nicht verstehe, lese ich sie mir mehrmals besonders langsam und konzentriert durch, um die (Fehler-)meldung zu verstehen. |  |
| If I did not understand everything in a meeting, I make sure to get all the required information. | Wenn ich in einer Besprechung nicht alles verstanden habe, stelle ich sicher, dass ich die nötigen Informationen bekomme. |  |
| Even though, with my function, I get nowhere in a task, I do not change my course of action. | Auch wenn ich mit meiner Arbeitsweise bei einer Aufgabe nicht vorankomme, ändere ich mein Vorgehen nicht. <REVERSED> |  |
| **Help Seeking** |  |  |
| I discuss specific problems concerning tasks and how to solve them with my superior. | Ich bespreche mit meinem Vorgesetzten, wie wir bestimmte Probleme in Bezug auf meine Arbeitsaufgaben lösen können. | X |
| If I do not understand something at work, I ask a colleague for help. | Wenn mir in meinem Arbeitsalltag etwas nicht klar ist, frage ich einen Arbeitskollegen um Rat. | X |
| If I have a problem that I cannot solve by myself, I know whom I can ask for help. | Wenn ich vor einem Problem stehe, das ich alleine nicht lösen kann, weiß ich, an wen ich mich wenden kann. | X |
| If problems occur at work, I use the intranet and/ or the internet to solve them. | Wenn auf der Arbeit Probleme auftreten, nutze ich das Intranet und/oder Internet, um diese zu lösen. |  |
| I do not look up information in user manuals or something similar, even if problems or problem reports occur that I do not know. | Ich schaue nicht in Benutzerhandbücher, Anleitungen (Arbeitsschritt-/Klickanleitungen) oder Ähnliches - selbst wenn sich Probleme oder Fehlermeldungen ergeben, die ich nicht kenne. <REVERSED> |  |
| I keep up-to-date with new findings concerning my work. | Ich bleibe auf dem Laufenden, ob es neue Erkenntnisse gibt, die für meine Arbeit relevant sind. |  |
| **Effort Regulation** |  |  |
| I work hard, even if I’m not good at doing the task. | Ich strenge mich auch dann an, wenn die Aufgabe mir gar nicht liegt |  |
| I do not give up, even if a task is very difficult. | Ich gebe nicht auf, auch wenn eine Aufgabe sehr schwierig ist. | x |
| When I set a time limit for specific tasks, I try to meet the deadline. | Wenn ich mir eine Frist für bestimmte Aufgaben gesetzt habe, versuche ich diese möglichst einzuhalten. |  |
| I give up easily if new tasks are difficult for me. | Bei neuen Aufgaben, die ich schwierig finde, gebe ich schnell auf. <REVERSED> | x |
| I try to finish tasks with as little effort as possible. | Ich versuche Aufgaben mit möglichst geringem Aufwand zu erledigen. <REVERSED> |  |

Table S2.

*SRL-questionnaire: preliminary items and self-translated preliminary items.*

| **Self-translated preliminary items** | **Preliminary Items** |
| --- | --- |
| **Organization** |  |
| When a colleague explains the application of a new system, or something similar, to me, I take notes that I can revise before I use the system. | Wenn mir ein Kollege die Anwendung eines neuen Systems oder Ähnliches erklärt, mache ich mir Notizen, die ich vor der tatsächlichen Anwendung nochmals durchgehen kann. |
| I create short work or click instructions for my tasks, to know how to operate in future situations. | Ich erstelle kurze Arbeitsschritt-/Klickanleitungen für meine Tätigkeiten, um beim nächsten Mal zu wissen, wie ich vorgehen muss. |
| Before I start a new task, I get an overview of the current conditions and make sure that I have all information and material needed. | Bevor ich eine neue Aufgabe beginne, verschaffe ich mir einen Überblick über die vorliegenden Bedingungen und stelle sicher, dass ich alle nötigen Informationen und Materialien dafür habe. |
| In general meetings I take notes of the most important points, so that I can use them for my work later. | In allgemeinen Besprechungen notiere ich mir die wichtigsten Punkte, damit ich sie später für meine Tätigkeit nutzen kann. |
| I use charts, tables or graphic illustrations in order to facilitate handling my task. | Ich nutze Diagramme, Tabellen oder grafische Darstellungen um mir die Bearbeitung meiner Aufgabe/ Tätigkeit zu erleichtern. |
| **Elaboration** |  |
| While I learn e.g the application of a new system, I try to connect this knowledge with my previous knowledge. | Während ich mir z.B. die Anwendung eines neuen Systems aneigne, versuche ich dieses Wissen mit meinem bisherigen Wissen zu verknüpfen. |
| When I get new professional information from my supervisor, I try to connect this new knowledge with my previous knowledge. | Wenn ich von meinem Vorgesetzten neue Fachinformationen bekomme, versuche ich das neue Wissen mit meinem bisherigen zu verbinden. |
| When I work on a new task, I try to bundle as much information as possible to help me. | Wenn ich eine neue Aufgabe bearbeite, versuche ich alles an Informationen zu bündeln, was mir weiterhilft. |
| When I’m facing a new task or problem, I draw back on previous experience to solve it. | Wenn ich vor einer neuen Aufgabe oder Problem stehe, greife ich auf meine bisherigen Erfahrungen zurück um sie zu lösen. |
| I try to create connections between different information I get during a da (e.g. from my supervisor, colleagues, etc.) | Ich versuche Zusammenhänge zwischen den verschiedenen Informationen, die ich im Arbeitsalltag bekomme (Z.B. über Vorgesetzte, Kollegen, etc.) herzustellen. |
| **Planning** |  |
| I usually keep track of the tasks that need to be done and prioritize them. | Ich behalte in der Regel den Überblick über die zu erledigenden Aufgaben priorisiere diese. |
| I do not plan my course of action at work. | Ich plane mein Vorgehen beim Arbeiten nicht. |
| When I start my work, I do not think about how to execute specific tasks. | Ich mache mir zu Arbeitsbeginn keine Gedanken, welche Aufgaben ich wie erledige. |
| I set daily or weekly goals. | Ich setze mir Tages- oder Wochenziele. |
| I define for myself, when I want to have specific tasks finished. | Ich lege für mich fest, bis wann ich welche Aufgaben erledigt haben möchte. |
| **Monitoring** |  |
| I check which operational steps I can carry out well and where I could improve myself. | Ich achte darauf, welche Arbeitsschritte ich gut ausführen kann und bei welchen ich mich noch verbessern könnte. |
| I regularly make myself aware of the areas where I have improved my skills in and the areas I’m still in need of development. | Ich mache mir regelmäßig bewusst, in welchen Bereichen ich mich verbessert habe und in welchen ich weiterhin Entwicklungsbedarf habe. |
| I notice when my thoughts get carried away and I’m not focused. | Mir fällt auf, wenn ich mit meinen Gedanken abschweife und nicht ganz bei der Sache bin. |
| Before I finish a task, I do not examine if I carried it out correctly / if it is error-free. | Bevor ich eine Aufgabe abschließe, prüfe ich nicht mehr, ob ich diese korrekt ausgeführt habe/ ob diese fehlerfrei ist. |
| After I finish a task, I examine if my proceedings were reasonable. | Nachdem ich eine Aufgabe abschließe, prüfe ich ob mein Vorgehen dabei sinnvoll war. |
| I regularly check, if my actions are still in line with the current conditions and constructive. | Ich achte regelmäßig darauf, ob meine Vorgehensweise noch den Bedingungen entsprechend und zielführend ist. |
| **Regulation** |  |
| When I’m assigned to a new task, I adapt my function to finish it at its best. | Wenn ich eine neue Aufgabe bekomme, passe ich meine Arbeitsweise an, um diese bestmöglich fertigzustellen. |
| When I find myself losing attention, I try to focus on the task again. | Wenn ich mich dabei ertappe, dass meine Aufmerksamkeit nachlässt, versuche ich mich wieder auf die Aufgabe zu fokussieren. |
| When I get a (problem) report e.g. on the computer, and I do not understand it, I read it again slowly and focused to understand the problem. | Wenn ich eine (Fehler-)meldung, z.B. am Computer, bekomme und sie nicht verstehe, lese ich sie mir mehrmals besonders langsam und fokussiert durch, um das Problem zu verstehen. |
| If I did not understand everything in a meeting, I ask for it or make sure that I will obtain and understand the information after the meeting. | Wenn ich in einer Besprechung nicht alles verstanden habe, frage ich nach oder stelle sicher, dass ich die Informationen nach dem Meeting einhole und verstehe. |
| Even though I get nowhere in a task with my function, I do not change my course of action. | Auch wenn ich mit meiner Arbeitsweise bei einer Aufgabe nicht vorankomme, ändere ich mein Vorgehen nicht. |
| **Help seeking** |  |
| I discuss specific problems concerning our tasks and how to solve them with my colleagues. | Ich diskutiere mit meinen Kollegen, wie wir bestimmte Probleme in Bezug auf unsere Arbeitsaufgaben lösen können. |
| If I do not understand something at work, I ask a colleague for help. | Wenn mir in meinem Arbeitsalltag etwas nicht klar ist, frage ich einen Arbeitskollegen um Rat. |
| If I have a problem that I cannot solve by myself, I know whom I can ask for help. | Wenn ich vor einem Problem stehe, das ich alleine nicht lösen kann, weiß ich an wen ich mich wenden kann. |
| I use the intranet and /or internet to gain information about procedures or problems at work. | Ich nutze das Intranet und / oder Internet, um Informationen zu Vorgehensweisen oder Problemen während der Arbeit zu erhalten. |
| I do not look up information in user manuals, instructions (work or click instrucitions) or something similar, even If problems or problem reports occur that I do not know. | Ich schaue nicht ins Benutzerhandbuch, Anleitungen (Arbeitsschritt-/ Klickanleitungen) oder Ähnliches – selbst wenn sich Probleme oder Fehlermeldungen ergeben, die ich nicht kenne. |
| I keep up-to-date with new findings concerning my work. | Ich bleibe auf dem Laufenden, ob es neue Erkenntnisse gibt, die für meine Arbeit relevant sind. |
| **Effort Regulation** |  |
| I work hard, even if I’m not good at doing the task. | Ich strenge mich auch dann an, wenn die Aufgabe mir gar nicht liegt. |
| I do not give up, even if a task is very difficult. | Ich gebe nicht auf, auch wenn eine Aufgabe sehr schwierig ist. |
| When I set a time limit for specific tasks, I try to meet the deadline. | Wenn ich mir eine Frist für bestimmte Aufgaben gesetzt habe, versuche ich diese möglichst einzuhalten. |
| I give up easily or let my colleagues do my work, if new tasks are difficult for me. | Bei neuen Aufgaben, die ich schwierig finde, gebe ich schnell auf oder lasse sie von meinen Kollegen bearbeiten. |
| I try to finish tasks with as little effort as possible. | Ich versuche Aufgaben mit möglichst geringem Aufwand zu erledigen. |

Table S3.

*Concrete wording of the PALS (Midgley et al., 2000) and the adapted and self-translated German items.*

| Original items | Adapted items | Adapted and self-translated items |
| --- | --- | --- |
| One of my goals in class is to learn as much as I can. | One of my goals at work is to learn as much as I can. | Eines meiner Ziele auf der Arbeit ist es, so viel wie möglich zu lernen. |
| One of my goals is to master a lot of new skills this year. |  | Eines meiner Ziele ist es, dieses Jahr viele neue Fähigkeiten zu erlernen. |
| It’s important to me that I improve my skills this year. |  | Es ist mir wichtig, meine Fähigkeiten dieses Jahr zu verbessern. |

Table S4.

*Concrete wording of* *short version of the Generalized Self-Efficacy Scale (Schwarzer & Jerusalem, 1999) to assess self-efficacy. As the CFA with ten original items revealed a poor fit with partly low loadings (≤ .4), we excluded the four items with loadings below ≤ .4.*

| Self-translated items | Reason to drop this item: | Original item |
| --- | --- | --- |
| When resistance arises, I find ways and means to assert myself. | Low loading; Focus on resistance, | Wenn sich Widerstände auftun, finde ich Mittel und Wege, mich durchzusetzen. |
| I always succeed in solving difficult problems if I try. | Low loading, focus on problem solving | Die Lösung schwieriger Probleme gelingt mir immer, wenn ich mich darum bemühe. |
| I have no difficulty in realizing my intentions and goals. | Low loading, focus on realizing intentions | Es bereitet mir keine Schwierigkeiten, meine Absichten und Ziele zu verwirklichen. |
| I always know how to act in unexpected situations. | Low loadings, focus on unexpected situations | In unerwarteten Situationen weiß ich immer, wie ich mich verhalten soll. |
| Even in the case of surprising events, I believe that I can get along well with them. |  | Auch bei überraschenden Ereignissen glaube ich, dass ich gut mit ihnen zurechtkommen kann. |
| I face difficulties calmly because I can always trust my abilities. |  | Schwierigkeiten sehe ich gelassen entgegen, weil ich meinen Fähigkeiten immer vertrauen kann. |
| Whatever happens, I'll be fine. |  | Was auch immer passiert, ich werde schon klar kommen. |
| I can find a solution for every problem |  | Für jedes Problem kann ich eine Lösung finden. |
| When a new thing comes my way, I know how to deal with it. |  | Wenn eine neue Sache auf mich zukommt, weiß ich, wie ich damit umgehen kann. |
| When I have a problem, I usually have several ideas on how to solve it. |  | Wenn ein Problem auf mich zukommt, habe ich meist mehrere Ideen, wie ich es lösen kann. |

Table S5.

*Concrete wording of the DLOQ (Watkins & Marsick, 1997) and the used self-translated German items.*

| Original items | Self-translated items |
| --- | --- |
| In my organization, people are rewarded for learning. | In meinem Unternehmen wird man fürs Lernen belohnt. |
| In my organization, people spend time building trust with each other. | In meinem Unternehmen verbringen die Menschen Zeit damit Vertrauen zu einander aufzubauen. |
| In my organization, teams/groups revise their thinking as a result of group discussions or information collected. | In meinem Unternehmen werden Ansichten innerhalb der Teams oder Gruppen gemeinsam mithilfe von Gruppendiskussionen oder gesammelter Informationen verändert. |
| My organization makes its lessons learned available to all employees. | Mein Unternehmen macht die „lessons learned“ (wichtige Erfahrungen, die für zukünftige Arbeitsprozesse berücksichtigt werden sollten) für alle Mitarbeiter zugänglich. |
| My organization recognizes people for taking initiative. | Mein Unternehmen würdigt Personen, die die Initiative ergreifen. |
| My organization works together with the outside community to meet mutual needs. | Mein Unternehmen kooperiert mit externen Partnern, um gemeinsame Ziele zu erreichen. |
| In my organization, leaders continually look for opportunities to learn. | In meinem Unternehmen suchen Führungskräfte fortwährend nach Möglichkeiten etwas Neues zu lernen. |
